# Screening performance of abbreviated versions of the UPSIT smell test

**DOI:** 10.1007/s00415-019-09340-x

**Published:** 2019-05-03

**Authors:** Theresita Joseph, Stephen D. Auger, Luisa Peress, Daniel Rack, Jack Cuzick, Gavin Giovannoni, Andrew Lees, Anette E. Schrag, Alastair J. Noyce

**Affiliations:** 1 University College London Medical School, London, UK; 2 Preventive Neurology Unit, Wolfson Institute of Preventive Medicine, Barts and the London School of Medicine and Dentistry, Queen Mary University of London, London, EC1M 6BQ UK; 3 Barts and The London School of Medicine and Dentistry, London, UK; 4 Blizard Institute, Barts and the London Queen Mary University of London, London, UK; 5 Reta Lila Weston Institute, Department of Clinical and Movement Neurosciences, UCL Institute of Neurology, London, UK; 6 Department of Clinical and Movement Neurosciences, UCL Institute of Neurology, London, UK

**Keywords:** Hyposmia, Parkinson’s disease, UPSIT, Smell tests, PREDICT-PD

## Abstract

**Background:**

Hyposmia can develop with age and in neurodegenerative conditions, including Parkinson’s disease (PD). The University of Pennsylvania Smell Identification Test (UPSIT) is a 40-item smell test widely used for assessing hyposmia. However, in a number of situations, such as identifying hyposmic individuals in large populations, shorter tests are preferable.

**Methods:**

We assessed the ability of shorter UPSIT subsets to detect hyposmia in 891 healthy participants from the PREDICT-PD study. Shorter subsets included Versions A and B of the 4-item Pocket Smell Test (PST) and 12-item Brief Smell Identification Test (BSIT). Using a data-driven approach, we evaluated screening performances of 23,231,378 combinations of 1–7 smell items from the full UPSIT to derive “winning” subsets, and validated findings separately in another 191 healthy individuals. We then compared discriminatory UPSIT smells between PREDICT-PD participants and 40 PD patients, and assessed the performance of “winning” subsets containing discriminatory smells in PD patients.

**Results:**

PST Versions A and B achieved sensitivity/specificity of 76.8%/64.9% and 86.6%/45.9%, respectively, while BSIT Versions A and B achieved 83.1%/79.5% and 96.5%/51.8%. From the data-driven analysis, 2 “winning” 7-item subsets surpassed the screening performance of 12-item BSITs (validation sensitivity/specificity of 88.2%/85.4% and 100%/53.5%), while a “winning” 4-item subset had higher sensitivity than PST-A, -B, and even BSIT-A (validation sensitivity 91.2%). Interestingly, several discriminatory smells featured within “winning” subsets, and demonstrated high-screening performances for identifying hyposmic PD patients.

**Conclusion:**

Using abbreviated smell tests could provide a cost-effective means of large-scale hyposmia screening, allowing more targeted UPSIT administration in general and PD-related settings.

**Electronic supplementary material:**

The online version of this article (10.1007/s00415-019-09340-x) contains supplementary material, which is available to authorized users.

## Introduction

A reduced ability to detect and recognise smells (hyposmia) commonly develops with increasing age [1], and can occur in otherwise healthy individuals as a result of head trauma, viral diseases including upper respiratory tract infections, sinusitis, or from inhalation of toxic fumes [1, 2]. In addition, hyposmia is increasingly recognised as an early feature of several age-related neurodegenerative disorders, including Parkinson’s disease (PD) [2, 3]. Indeed, hyposmia is observed in up to 90% of PD patients [4], and is considered a sensitive non-motor symptom for discriminating between PD patients and healthy controls [5]. The onset of hyposmia is associated with an increased risk of being diagnosed with PD [6–8], and can predate motor symptoms by years [9, 10]. The neural substrate behind olfactory dysfunction in PD is incompletely understood; however, neuropathological evidence points to the olfactory bulb being among the first regions to demonstrate neuronal loss and accumulation of intracytoplasmic a-synuclein rich Lewy bodies [11–13], before the pathology involves more central regions. Thus, olfactory dysfunction is increasingly recognised as a potential marker for the early identification of neurodegenerative processes [14–16].

Several smell tests have been created to screen for olfactory dysfunction, including tests of odour adaptation, discrimination, detection, identification, memory, and suprathreshold intensity scaling [17]. The University of Pennsylvania Smell Identification Test (UPSIT), marketed by Sensonics International as the Smell Identification Test, is one of the most commonly-used smell tests worldwide [18], and comprises 40 “scratch-and-sniff” microencapsulated odorant strips divided across 4 booklets (10 in each). For each strip, participants are required to identify the correct smell from a forced choice of 4 possible answers. The total number of smells correctly identified out of 40 is then compared with normative age- and sex-specific thresholds for olfactory dysfunction [18]. Its popularity reflects its ability to be self-administered, to differentiate among different levels of less-than-total dysfunction, and to detect malingering.

Shorter smell identification tests have also been developed, either as standalone [versions of 12-item Brief Smell Identification Test (BSIT)] or preliminary tests [versions of 4-item Pocket Smell Test (PST)], to guide later administration of the UPSIT to relevant individuals (see Supplementary Table 1). A comprehensive list of these and other smell tests developed have been reviewed elsewhere [19]. Overall, these tests provide utility in the general assessment of olfactory dysfunction, and some have shown sensitivity for certain neurodegenerative diseases [20, 21]. However, to date, there are no smell tests which can confirm the aetiology of particular cause of olfactory dysfunction.

In this study, we examined the screening performance of the current 4-item PSTs and 12-item BSITs in a large group of healthy, older individuals from the PREDICT-PD study, and assessed the tests’ ability to detect hyposmia according to the full 40-item UPSIT. We then sought to identify novel subset(s) of UPSIT items with superior predictive capabilities in the same group, and validated the findings in an independent group of individuals from the same study. We hypothesised that smells from these “winning” subsets could be used as a more accurate and cost-effective pre-screening tool for olfactory dysfunction, and thus assessed certain “winning” subsets on their performance in detecting hyposmia in individuals with PD.

## Methods

### Participant details

**Fig 1:**
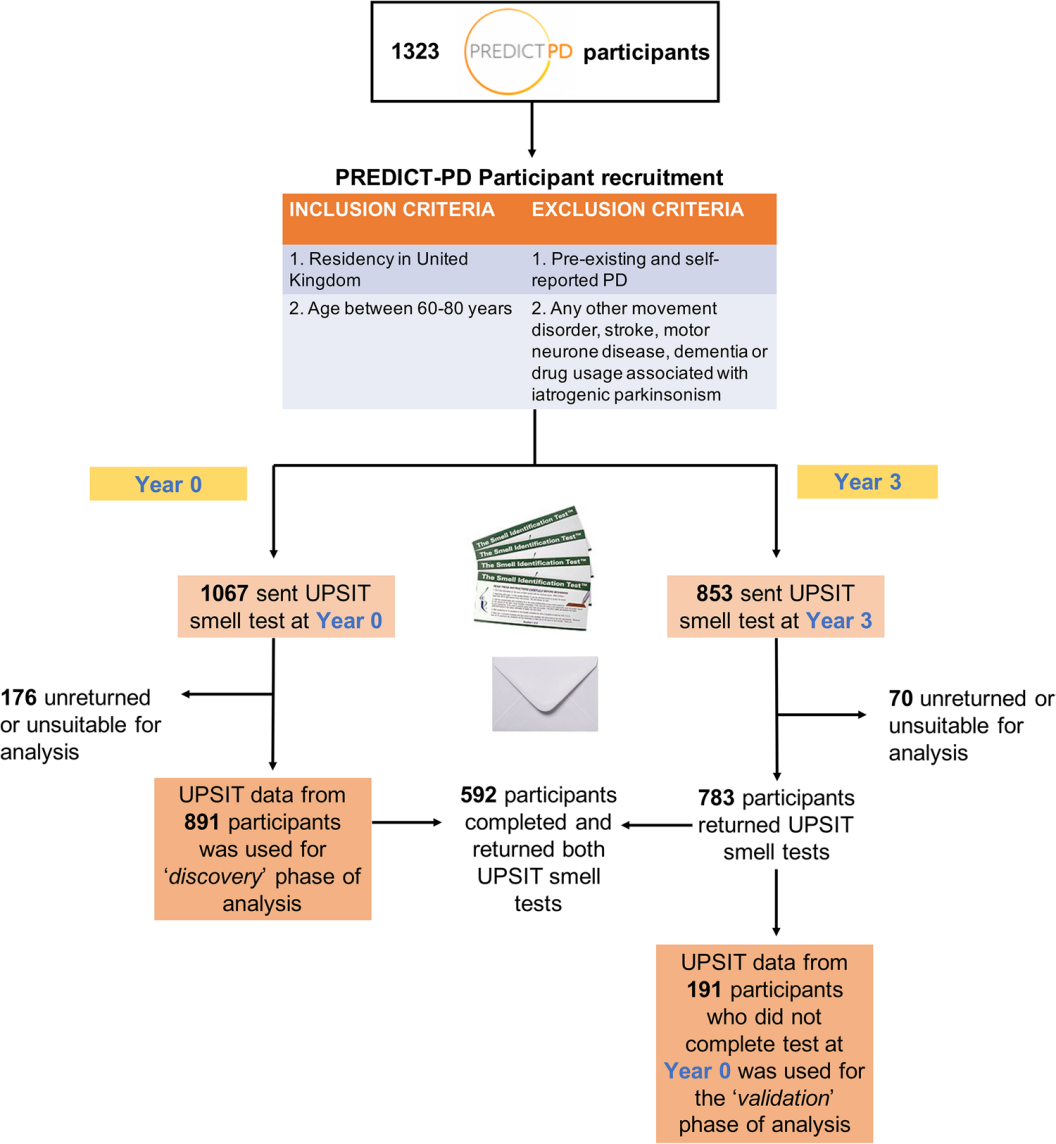
Schematic workflow of PREDICT-PD participation in year 0 and year 3 and where UPSIT data were available for ‘discovery’ and ‘validation’ analysis

We used data from the PREDICT-PD cohort, a study of 1323 individuals recruited from the general population in the UK between the ages of 60–80. Details of recruitment into the PREDICT-PD study have been described elsewhere [22]. Of the 1067 participants from the PREDICT-PD cohort who were sent the full 40-item US version of the UPSIT in the baseline year of the study, 891 completed the test that year (mean age 67.3 years, SD 4.8, 61.5% female). A group of 191 participants who completed the UPSIT test in only year 3 of the study were used for the validation of “winning” smell subsets (mean age 69.8 years, SD 4.7, 61.8% female). Figure 1 outlines the workflow of UPSIT data collection from the PREDICT-PD study.

A separate group of 40 individuals with established PD, who were positive controls for the PREDICT-PD study (mean age 63.8, SD 9.6, 25% female) were also sent and returned the full 40-item UPSIT.

### Assessment of current abbreviated smell tests

Shorter smell tests marketed by Sensonics International include Versions A and B of the 4-item PST. A test subject is recommended to undergo full UPSIT testing if they cannot correctly identify 1 or more smells in either PST version. The original selection of smells in each version of the PST was based upon their relevance to diet and nutrition, household, and public safety, rather than empirical evidence relating to smell identification [23]. Of the abbreviated standalone tests for olfactory dysfunction by the same company, the BSIT is a validated, cross-cultural 12-item version of the UPSIT [24]. Notably, the smells and response alternatives in BSIT Versions A and B have shown to possess some discriminatory power for specific neurodegenerative diseases according to the studies on which they were based; BSIT-A for AD [20], and BSIT-B for PD [21], although they do not confirm a diagnosis of either disease [19].

Scores for all 40 UPSIT smells were recorded for each participant. ‘Hyposmia’ was defined as being the lowest 15th centile of UPSIT scores according to age and sex (in 5-year bins). This method was used over pre-set threshold scores defined within the UPSIT administration manual, given that these thresholds were derived from a US population and the current study was undertaken in UK participants. Participants with smell scores below the hyposmic thresholds included those who were anosmic (i.e., had complete smell loss). Screening performance of each abbreviated smell test for hyposmia detection was assessed against the corresponding total UPSIT scores for each participant. For the 4-item PST and 12-item BSIT versions, scores of ≤ 3 and ≤ 9, respectively, were indicative of a positive hyposmia screen. Sensitivity, specificity, positive predictive value (PPV). and negative predictive value (NPV) were calculated for each test.

### Data-driven approach to identify novel optimal smell subsets

The discovery phase for novel smell subsets was undertaken using data from the 891 healthy participants (discovery cohort) and assessed all 23,231,378 possible combinations of 1–7 smells from the total of 40 UPSIT smells. For each smell combination, the ability to detect hyposmia was assessed against the full UPSIT score, and was defined in terms of sensitivity, specificity, PPV and NPV, as well as different score thresholds for defining hyposmia. For example, for each of the 18,643,560 combinations of 7 smell subsets from the full set of 40, we assessed screening performance based upon hyposmia being defined as participants scoring 0/7, ≤ 1/7, ≤ 2/7, ≤ 3/7, ≤ 4/7, ≤ 5/7, and ≤ 6/7. The different thresholds for each combination of 7 smell subsets meant that we assessed 130,504,920 sets of smell combinations and hyposmia thresholds. Combining this with the same approach for 1–6 smell subsets led to the assessment of a total 157,222,040 possible screening tests.

A “winning” subset of smells at each hyposmia threshold was selected according to those with the highest combined sensitivity and specificity. For example, when considering 5 smell items at a threshold of ≤ 4 to define hyposmia, sensitivity was the number of people who both correctly identified ≤ 4 of the 5 smells and were defined as hyposmic according to the full UPSIT, divided by the total number of hyposmic participants according to the full UPSIT. Specificity was the number of people who correctly identified all 5 smells in the subset and were not hyposmic according to the full UPSIT, divided by all those who were not hyposmic as defined by the full UPSIT. These two values were then summed and the combination of smells with the highest combined value was deemed to be the “winner” for that specific threshold. The same process was repeated for every threshold of hyposmia, for all numbers of smell combinations.

Using this method, rather than the area under curve (AUC), allowed us to identify the best performing combinations of smells across all possible thresholds, rather than one which performed best when averaging across a number of thresholds (as an AUC would). Hence, it allowed us to identify threshold-specific optimal smell subsets and enable comparison of different hyposmia thresholds.

The screening performance of each “winning” subset was reassessed in an independent group of 191 healthy PREDICT-PD participants (validation cohort). There was no overlap in the participants included for selecting the “winning” subsets and the subsequent testing of them (Fig. 1). Therefore, the results reported are more likely to be generalisable and not due to overfitting of the model.

### Validation of the novel smell subsets in individuals with PD

We then evaluated smell identification in the context of PD. We compared the proportion of smells correctly identified by 40 individuals with PD and the healthy PREDICT-PD participants for all 40 UPSIT items. For the top 10 smells with the largest difference in correct identification between individuals with PD and healthy controls, we looked at how commonly these featured in our “winning” smell subsets from the previous phase of the analysis. “Winning” smell subsets containing at least 2 of the top 10 discriminatory smells were subsequently assessed for their screening performance in detecting hyposmia in the same individuals with PD, compared to currently available PST and BSIT tests.

## Results

Based on total UPSIT scores and age- and sex-specific thresholds of PREDICT-PD participants, 16.2% females (89/548) and 16.0% males (55/343) from the 891 participants in the discovery cohort were classified as having hyposmia. Smoke was the most common correctly identified smell (851/891), and turpentine the least common correctly identified smell (328/891). Of the 191 validation cohort participants, 13.6% females (16/118) and 24.7% males (18/73) were hyposmic. Amongst the 40 PD participants who were sent and completed the UPSIT, 70% females (7/10) and 83.3% males (25/30) were hyposmic, in keeping with the known higher prevalence of hyposmia in PD patients compared to healthy participants [3].

### PST and BSIT hyposmia screening performance

Table 1 displays the screening performances of abbreviated smell tests assessed in the discovery cohort. Using the recommended cut-off score of ≤ 3 correctly identified smells to denote hyposmia, PST Version A detected hyposmia with sensitivity 76.8%, specificity 64.9%, PPV 29.3%, and NPV 93.6%. PST Version B had a greater sensitivity 88.6% and NPV 94.8%, but lower specificity 45.9% and PPV 23.3%. For the 12-item BSITs, the standard cut-off score of ≤ 9 on BSIT-A detected hyposmia with a sensitivity of 83.1%, specificity 79.5%, PPV 43.5%, and NPV 96.1%. Comparatively, BSIT-B had greater sensitivity 96.5% and NPV 98.7% than BSIT-A, but less specificity 51.8% and PPV 27.5%. We also assessed different score thresholds of the BSIT, which are presented in full in Supplementary Tables 2 and 3*.*

**Table 1:** Screening performance of PST and BSIT Versions A and B for hyposmia detection in discovery cohort compared against the UPSIT

Shorter test version	Number of smells	Hyposmia score	Sensitivity (%)	Specificity (%)	PPV (%)	NPV (%)
PST Version A	4	≤ 3	76.8	64.9	29.3	93.6
PST Version B	4	≤ 3	86.6	45.9	23.3	94.8
BSIT-A	12	≤ 9	83.1	79.5	43.5	96.1
BSIT-B	12	≤ 9	96.5	51.8	27.5	98.7

*PPV *positive predictive value, *NPV *negative predictive value

### Identifying optimal smell subsets

We next assessed all combinations of 1–7 smells from the full set of 40 UPSIT smells in the discovery cohort, from which there was a total of 28 “winning” smell combinations. Table 2 shows a selection of these “winning” smell combinations and the threshold scores for defining hyposmia. The sensitivity, specificity, PPV, and NPV values shown are from their assessment in both discovery and validation cohorts. The complete results from the data-driven analysis with all 28 “winning” smell combinations at each threshold are presented in full in Supplementary Tables 4 and 5, showing their screening performance in the discovery and validation cohorts, respectively.

**Table 2:** Screening performance of selected “winning” smell subsets from discovery cohort and reassessment in validation cohort compared against the UPSIT

No. of smells	Hyposmia cut-off score	Smells “winning” in discovery cohort	Sens. in discovery cohort	Spec. in discovery cohort	PPV in discovery cohort	NPV in discovery cohort	Sens. in validation cohort	Spec. in validation cohort	PPV in validation cohort	NPV in validation cohort
1	0	Pizza	69.7	66.1	28.0	92.0	82.4	53.5	27.7	93.3
2	≤ 1	Clove, Coconut	62.0	88.7	50.9	92.5	61.8	82.8	43.8	90.9
3	≤ 1	Pizza, Clove, Root beer	72.5	79.3	39.9	93.8	70.6	80.9	44.4	92.7
4	≤ 2	Cherry, Clove, Coconut, Root beer	72.5	87.2	51.8	94.4	61.8	88.5	53.8	91.4
4	≤ 3	Menthol, Clove, Gingerbread, Orange	78.9	82.8	46.5	95.4	91.2	35.0	23.3	94.8
5	≤ 2	Pizza, Clove, Fruit punch, Chocolate, Root beer	71.8	88.4	54.0	94.3	73.5	84.1	50.0	93.6
5	≤ 4	Menthol, Clove, Onion, Gingerbread, Orange	83.1	80.4	44.5	96.2	94.1	33.8	23.5	96.4
6	≤ 3	Pizza, Clove, Fruit punch, Liquorice, Lime, Pine	83.8	82.6	47.8	96.4	85.3	78.3	46.0	96.1
6	≤ 4	Menthol, Cherry, Clove, Gingerbread, Root beer, Orange	84.5	83.8	49.8	96.6	88.2	65.6	35.7	96.3
7	≤ 4	Cherry, Mint, Clove, Fruit punch, Gingerbread, Root beer, Pine	82.4	87.7	56.0	96.3	88.2	85.4	56.6	97.1
7	≤ 5	Pizza, Menthol, Cherry, Clove, Gingerbread, Orange, Pine	87.3	83.0	49.4	97.2	100.0	53.5	31.8	100.0

*Sens. *sensitivity, *Spec.* specificity, *PPV *positive predictive value, *NPV *negative predictive value

Table 2 reveals that the “winning” smell subsets have different relative strengths in terms of sensitivity, specificity, PPV, and NPV. In both discovery and validation cohorts, optimised combinations of 7 smell items showed superior screening performance to the 12-item BSITs. 7 smells using a cutoff of 4 for hyposmia surpassed the sensitivity/specificity of BSIT-A (88.2/85.4 vs 83.1/79.5) and another 7 smells with a hyposmia cutoff of 5 surpassed that of the more sensitive BSIT-B (100/53.5 vs 96.5/51.8). Using as few as 6 smell items produced comparable screening performance to the 12-item BSIT (sensitivity/specificity for 6 smells with a cutoff of 3: 85.3/78.3 vs 83.1/79.5 for BSIT-A). Following acquisition of these results, further analysis of smell combinations using > 7 UPSIT items was deemed unnecessary and was not pursued.

For the purpose of designing a short pre-screening smell test, which would best identify individuals who require further smell testing, it was important to maximise sensitivity and NPV to minimise the number of impaired individuals excluded from further testing. In this regard, a 4-item subset (menthol, clove, gingerbread, orange) with a cutoff of 3 or less produced higher sensitivity and NPV scores in both discovery and validation cohorts when compared with both current 4-item PST tests **(**see Table 1, Supplementary Table 4 and Table 2).

### Comparison of smell identification between PD and healthy participants

**Fig. 2:**
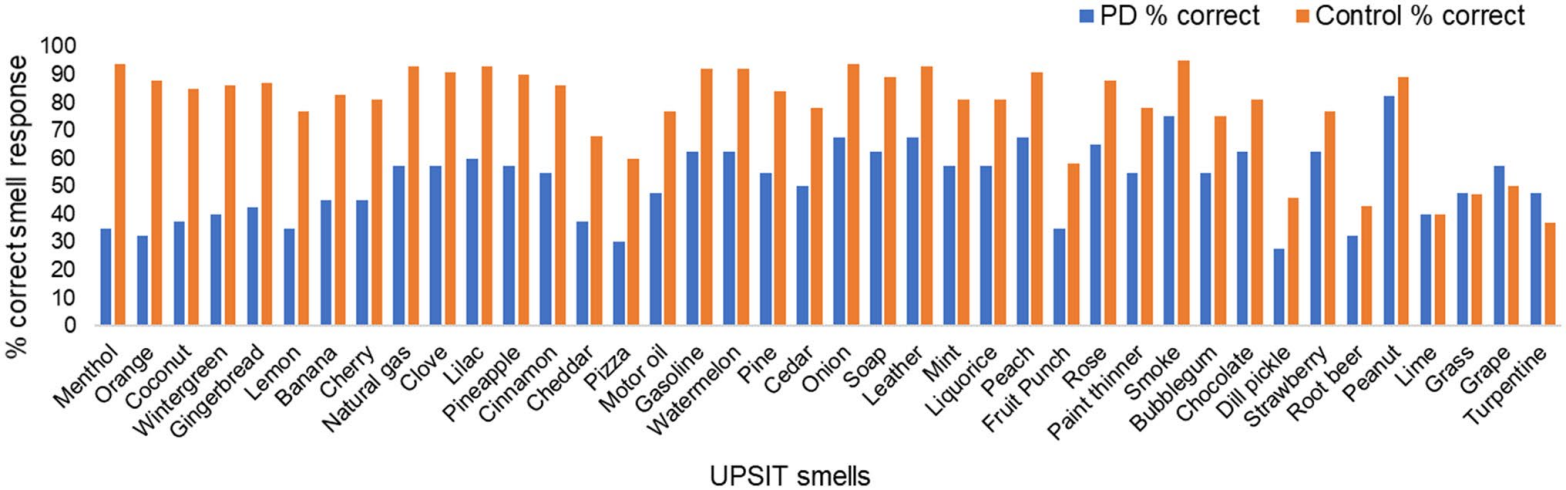
Identification rates of the 40 individual smells in the full UPSIT in 40 PD participants (blue) and 891 healthy PREDICT-PD controls (red). Smells are ordered by those with the greatest difference in correct smell identification by PD participants’ versus healthy controls on the left

The results from a comparison of correctly identified UPSIT smell items in the 40 patients with PD and the 891 healthy PREDICT-PD participants are shown in Fig. 2.

The most discriminating smells (i.e., those with the largest differences in correct identification between PD patients and PREDICT-PD controls) included menthol, orange, and coconut, while the least discriminating smells included turpentine, grape, and grass. Interestingly, several of the most discriminating smells frequently featured within the “winning” UPSIT combinations chosen for hyposmia detection in healthy individuals from the data-driven analysis (Supplementary Tables 4 and 5).

Based on these findings, we assessed the screening performance of “winning” smell subsets containing at least 2 of the top 10 discriminatory smells in accurately detecting hyposmia (by UPSIT) in the 40 PD patients within this study, compared to the performance of current PST and BSIT versions (see Table 3). Improved screening performance was observed in “winning” smell subsets that contained discriminating smells, with subsets containing ≥ 4 discriminating smells all having 100% sensitivity for detecting hyposmic PD patients. This included the more ‘PD-specific’ BSIT-B, which contained 5/10 top discriminatory smells. Significantly, the 4-item combination of menthol, clove, gingerbread and orange used in 4 and 5-item subsets had the highest performance score for both sensitivity and specificity (100% and 87.5%) than all other smell combinations, surpassing the performance of both 12-item BSITs. In addition, the use of the 2-item subset of clove and coconut had a higher/equal sensitivity to 4-item PST-B and PST-A, respectively. However, in some smell subsets, higher numbers of discriminating smells appeared to decrease specificity; for example, the specificity was reduced from 25% to 12.5% in the 4-item combination of cherry, clove, coconut and root beer, compared to the 2-item combination of clove and coconut.

**Table 3:** Screening performance for detecting hyposmia defined by the UPSIT in PD patients using “winning” smell subsets which contain at least 2 of the 10 most discriminating smell and current abbreviated smell tests

Total no. of smells	No. of discriminatory smells	Hyposmia cut-off score	Abbreviated smell test	Sensitivity (%)	Specificity (%)
4	4	≤ 3	**Menthol, Clove, Gingerbread, Orange**	100	87.5
5	4	≤ 4	**Menthol, Clove**, **Gingerbread, Orange**, Onion	100	87.5
12	5	≤ 9	BSIT-B (includes, **Clove, Coconut**, **Lemon**, **Wintergreen, Banana**)	100	62.5
7	5	≤ 5	**Menthol**, **Cherry**, **Clove**, **Gingerbread, Orange**, Pine, Pizza	100	37.5
6	5	≤ 4	**Menthol, Cherry**, **Clove, Gingerbread,orange,** Root beer	100	12.5
7	2	≤ 6	**Menthol, Clove,** Leather, Lilac, Watermelon, Smoke, Rose	96.9	37.5
6	3	≤ 5	**Menthol, Clove, Gingerbread**, Lilac, Watermelon, Smoke	96.9	25
3	3	≤ 2	**Menthol, Clove, Coconut**	96.9	25
5	4	≤ 3	**Menthol**, **Cherry**, **Clove, Coconut,** Root beer	96.9	12.5
7	3	≤ 4	**Cherry**, **Clove**, **Gingerbread**, Fruit punch, Root beer, Pine, Mint	96.9	12.5
12	2	≤ 9	BSIT-A (includes **Lemon, Banana**)	90.6	25
4	0	≤ 3	PST-A (none)	81.3	37.5
2	2	≤ 1	**Clove, Coconut**	81.3	25
4	3	≤ 2	**Cherry**, **Clove, Coconut,** Root beer	81.3	12.5
4	0	≤ 3	PST-B (none)	75	25

Smell subsets are ordered according to their respective sensitivities, then by specificity. The most discriminating smells are highlighted in bold

## Discussion

In the first part of this study, we assessed the screening performance of abbreviated UPSIT smell subsets for their ability to detect hyposmia within a large UK-based population of healthy individuals in the PREDICT-PD study. On assessment of the current commercially available BSIT and PST smell tests (Versions A and B), the 12-item BSITs had an expected greater screening performance for detecting hyposmia compared with either 4-item PST. This reflects the BSITs’ ability to act as standalone tests for hyposmia, whereas PSTs are intended as a pre-screen to target subsequent administration of full UPSIT testing. However, our results highlighted differences in the sensitivity and specificity of each 4-item PST, suggesting that the accuracy with which they can detect hyposmia varies depending on the version administered, which should be taken into consideration with future use.

In our data-driven analysis, we identified novel UPSIT subsets of just 7 smell items that had superior screening performance compared to the 12-item BSITs for detecting hyposmia in healthy PREDICT-PD individuals. Importantly, these “winning” smell subsets identified in the discovery phase appeared to retain their overall screening performance with independent testing in the validation phase. Using as few as 6 smell items could offer comparable screening performance to the BSIT. Given that these combinations are half the length of the current BSIT, expansion of their use could offer obvious benefits in terms of time and expense when undertaking large-scale studies, or for use in routine clinical settings.

We also identified a subset of 4 smells (menthol, clove, gingerbread, and orange) which had a high-screening performance in the discovery cohort, and identified a greater proportion of individuals with hyposmia than either Version A or B of the 4-item PST when reassessed in the independent validation group. While the above 7 and 6 item tests may be suitable for standalone testing, this optimised 4-item subset may be an ideal pre-screen test, before selective use of the UPSIT for assessing olfactory dysfunction in the general population. Nevertheless, it is still important to remember that while shorter test versions may provide an effective method for hyposmia screening, they do not have the added benefits that longer tests offer in being able to distinguish between levels of less-than-total olfactory dysfunction or for identifying malingering, hence should not be seen as a substitute for more extensive forms of smell testing.

In the context of assessing hyposmia in PD, studies have consistently demonstrated that PD patients have lower total UPSIT scores compared with healthy controls [25, 26], which was borne out in our results. In the second part of this study, we attempted to investigate whether any of our “winning” shorter smell subsets also had value in assessing hyposmia in patients with PD. Specifically, we assessed differences in correct smell identification responses between the 40 PD and 891 healthy PREDICT-PD individuals of this study to investigate for discriminatory smells. Interestingly, menthol, clove, gingerbread, and orange featured amongst the top 10 discriminatory smells, and when included within 4- and 5-item subsets correctly identified hyposmia in all of the 32 hyposmic patients with PD, as well as correctly classified 7 of the 8 normosmic patients with PD (sensitivity/specificity 100% and 87.5%), surpassing the screening performance of both 12-item BSITs. Further investigation into the utility of these smell subsets for hyposmia detection in larger PD cohorts would be of benefit. However, while the present study demonstrates the ability of certain smell subsets to detect hyposmia in individuals with PD (as well as the healthy population), it does not offer confirmatory evidence of there being a PD-specific patterns of olfactory dysfunction. Formal comparison with hyposmia due to other causes would be required to make claims as to such disease-specific detection.

Indeed, there has been extensive debate as to whether specific smells are lost preferentially over the course of PD. Some work suggests that specific smells can differentiate people with and without PD [27–29]; however, there is significant variability between which smells are implicated, while others have found no evidence of PD-specific smell loss [25, 20]. By example, a recent study attempting to devise novel UPSIT subsets specific for the detection of PD found subsets with good screening performance in discovery analyses, but failed to retain this performance when reassessed in independent groups [19]. Overall, these variable findings may be due to several confounding factors, including the choice of smell test, alternative smell (‘distractor’) options, study populations, and cultural differences.

Alternative methods to increase the screening performance of smell tests for identifying individuals at risk of PD have also been investigated. These methods include combination of smell scores with other early, nonmotor PD manifestations, such as constipation, sleep disturbances, and depression [16, 31]. Moreover, another recent study identified a PD-specific response pattern of 12 incorrect UPSIT question/response pairs in PD participants compared to healthy controls [32], which appeared more valuable for PD diagnosis than total mean UPSIT score. Assessing for this sort of disease-specific olfactory loss was beyond the scope of the present study, but would certainly merit further work in the future. Additional adaptations in the design of future smell tests could also include the use of confidence ratings into each answer panel, ranking from 1 (least confident) to 4 (very confident), to provide a greater yield of information over individuals’ identification of specific smells without lengthening the test.

A key strength of the present study is its size. To the best of our knowledge, this is the largest assessment of screening performance of abbreviated versions of smell identification tests in comparison with the full 40-item UPSIT. However, there are certain limitations. First, given that the assessment of all of abbreviated smell tests was based upon comparison with participants’ total UPSIT score, we are assuming that it still remains an accurate and sensitive tool for detecting hyposmia in the general healthy population as validated by Doty et al. [33]. The three ‘distractor’ options used in both PST versions may also differ from those for the same smells in the full UPSIT test (see Supplementary Table 6). These different distractors could have influenced participants’ ability to identify the smells to some degree, but the impact on overall screening performance assessed is likely to be relatively small, given that the target smells are the same. In the same way, given that our data-driven analysis of multiple UPSIT smell combinations was based on existing UPSIT data from participants, we acknowledge the potential influence of distractor options for each smell on the correct identification, and thus the ranking of “winning” smell subsets.

Another limitation is that the current study used the original US version of the UPSIT, but in a UK population, which might have lowered overall performance due to reduced familiarity with some of the smells. For example, the wintergreen smell is likely to be more familiar to an American population than in the UK. Indeed, a previous UK-based study using the US UPSIT found certain smells to have low identification rates [34]. The smells with the lowest cross-cultural detection included root beer (52.3%), lime (56.8%), dill pickle (61.4%), and turpentine (65.9%) [34], which was borne out in our own data as some of the poorest identified smells in healthy participants, as well as the worst discriminating smells between PD patients and healthy participants. Of note, turpentine and grape were the only two smells within our study that had a higher correct detection by PD patients than healthy participants. In light of these issues, “winning” subsets which only include smells present in the UK UPSIT version are provided in Supplementary Table 7.

Finally, while the current study identified individuals as ‘hyposmic’ based on specific age and sex threshold cutoffs within the PREDICT-PD population, the data-driven approach did not include age or sex in their parameters for assessment of “winning” USPIT smell subsets at each threshold cutoff. Given the established influence of age and sex on olfaction [2, 35], it is possible that their inclusion in the analysis could lead to further improvement in the screening performance of abbreviated subsets, and this will be evaluated in future work.

## Conclusion

Accurate assessment of olfactory dysfunction may assist in the early detection of certain neurodegenerative diseases such as PD. Using a data-driven approach, our study identified several “winning” 1–7 UPSIT smell subsets with high-screening performance for hyposmia detection in 891 healthy participants of the PREDICT-PD study. Of note, 7-item subsets demonstrated superior screening performance to current 12-item BSIT versions, which was retained on reassessment within an independent cohort. Our study also found that “winning” subsets containing smells which had large differences in correct identification rates between individuals with and without PD also produced high-screening performances when assessing for hyposmia in individuals with PD, including menthol, clove, gingerbread, and orange. Notably, several 3-, 4-, and 5-item subsets incorporating some of the top 10 discriminatory identified more PD patients with hyposmia than current PST and even BSIT-A test versions. Significant cost and efficiency savings may be gained using these smell combinations within an abbreviated smell test to target more focused administration of the full UPSIT for wider scale clinical and research purposes, in both general and PD-related settings.

## Electronic supplementary material

Below is the link to the electronic supplementary material.

Supplementary file1 (DOCX 32 kb)
