# Supplementary material for: Screening performance of abbreviated versions of the UPSIT smell test
Source: J Neurol. 2019 May 3;266(8):1897–906. doi: 10.1007/s00415-019-09340-x (PMC6647236; doi:10.1007/s00415-019-09340-x)
Supplement: Supplementary file 1 — Supplementary file1 (DOCX 32 kb) [file 415_2019_9340_MOESM1_ESM.docx]

**Supplementary Material**

**Supplementary Table 1:** Specific smells included in current smell tests developed by Sensonics International

| **Smell Test** | **Smells included** |
| --- | --- |
| 40 item UPSIT | pizza, bubblegum, menthol, cherry, motor oil, mint, banana, clove, leather, coconut, onion, fruit punch, liquorice, cheddar cheese, cinnamon, gasoline, strawberry, cedar, chocolate, ginger, lilac, turpentine, peach, root beer, dill pickle, pineapple, lime, orange, wintergreen, watermelon, paint thinner, grass, smoke, pine, grape, lemon, soap, natural gas, rose, peanut |
| 4-item PST Version A | chocolate, strawberry, smoke, leather |
| 4-item PST Version B | soap, grape, onion, natural gas |
| BSIT-A | banana, chocolate, cinnamon, gasoline, lemon, onion, paint thinner, pineapple, rose, soap, smoke, turpentine |
| BSIT-B | banana, clove, coconut, grass, lemon, liquorice, lime, paint thinner, pizza, rose, strawberry, wintergreen |

**Supplementary Tables 2 and 3:** Different score thresholds of BSIT-A **(Sup. Table 2)** and BSIT-B **(Sup. Table 3)** for detecting hyposmia in 891 PREDICT-PD baseline participants

**Supplementary Table 2:**

| **Number of smells** | **Hyposmia cutoff** | **Sensitivity** | **Specificity** | **PPV** | **NPV** |
| --- | --- | --- | --- | --- | --- |
| 12 | ≤1 | 0.7 | 100.0 | 100.0 | 84.2 |
| 12 | ≤2 | 2.1 | 100.0 | 100.0 | 84.3 |
| 12 | ≤3 | 4.9 | 100.0 | 100.0 | 84.7 |
| 12 | ≤4 | 7.0 | 100.0 | 100.0 | 85.0 |
| 12 | ≤5 | 9.9 | 100.0 | 100.0 | 85.4 |
| 12 | ≤6 | 20.4 | 99.9 | 96.7 | 86.9 |
| 12 | ≤7 | 33.8 | 98.3 | 78.7 | 88.7 |
| 12 | ≤8 | 62.7 | 94.1 | 66.9 | 93.0 |
| **12** | **≤9** | **83.1** | **79.6** | **43.5** | **96.1** |
| 12 | ≤10 | 96.5 | 50.5 | 27.0 | 98.7 |
| 12 | ≤11 | 98.6 | 15.4 | 18.1 | 98.3 |

**Supplementary Table 3:**

| **Number of smells** | **Hyposmia cutoff** | **Sensitivity** | **Specificity** | **PPV** | **NPV** |
| --- | --- | --- | --- | --- | --- |
| 12 | ≤1 | 0.7 | 100.0 | 100.0 | 84.2 |
| 12 | ≤2 | 2.8 | 100.0 | 100.0 | 84.4 |
| 12 | ≤3 | 13.4 | 100.0 | 100.0 | 85.9 |
| 12 | ≤4 | 19.7 | 100.0 | 100.0 | 86.8 |
| 12 | ≤5 | 30.3 | 99.6 | 93.5 | 88.3 |
| 12 | ≤6 | 49.3 | 97.7 | 80.5 | 91.0 |
| 12 | ≤7 | 73.9 | 91.9 | 63.3 | 94.9 |
| 12 | ≤8 | 90.8 | 78.8 | 44.8 | 97.8 |
| **12** | **≤9** | **96.5** | **51.8** | **27.5** | **98.7** |
| 12 | ≤10 | 100.0 | 24.8 | 20.1 | 100.0 |
| 12 | ≤11 | 100.0 | 6.1 | 16.8 | 100.0 |

*Note: Hyposmia cut off score of ≤9 (bold) is the current recommendation for BSIT test use.*

**Supplementary Tables 4 and 5:** Complete set of 28 “winning” subsets derived from data-driven analysis of 23,231,378 combinations of 1-7 UPSIT smell items in the *discovery* cohort of 891 PREDICT-PD participants **(Sup. Table 4)** and their re-assessment in the *validation* cohort 191 PREDICT-PD participants **(Sup. Table 5)**

**Supplementary Table 4:**

| **Number of smells** | **+ve* cutoff** | **Sensitivity** | **Specificity** | **PPV** | **NPV** | **Smells** |
| --- | --- | --- | --- | --- | --- | --- |
| 1 | 0 | 69.7 | 66.1 | 28.0 | 92.0 | Pizza |
| 2 | 0 | 59.2 | 80.8 | 36.8 | 91.3 | Pizza, Root beer |
| 2 | 1 | 62.0 | 88.7 | 50.9 | 92.5 | Clove, Coconut |
| 3 | 0 | 43.7 | 88.9 | 42.8 | 89.3 | Pizza, Root beer, Dill pickle |
| 3 | 1 | 72.5 | 79.3 | 39.9 | 93.8 | Pizza, Clove, Root beer |
| 3 | 2 | 71.8 | 86.8 | 50.7 | 94.2 | Menthol, Clove, Coconut |
| 4 | 0 | 33.1 | 94.0 | 51.1 | 88.1 | Pizza, Root beer, Lime, Grape |
| 4 | 1 | 66.2 | 83.8 | 43.7 | 92.9 | Pizza, Cherry, Turpentine, Root beer |
| 4 | 2 | 72.5 | 87.2 | 51.8 | 94.4 | Cherry, Clove, Coconut, Root beer |
| 4 | 3 | 78.9 | 82.8 | 46.5 | 95.4 | Menthol, Clove, Gingerbread, Orange |
| 5 | 0 | 24.6 | 96.9 | 60.3 | 87.2 | Pizza, Root beer, Lime, Grass, Grape |
| 5 | 1 | 54.2 | 91.1 | 53.5 | 91.3 | Pizza, Cherry, Turpentine, Root beer, Lime |
| 5 | 2 | 71.8 | 88.4 | 54.0 | 94.3 | Pizza, Clove, Fruit Punch, Chocolate, Root beer |
| 5 | 3 | 79.6 | 85.8 | 51.6 | 95.7 | Menthol, Cherry, Clove, Coconut, Root beer |
| 5 | 4 | 83.1 | 80.4 | 44.5 | 96.2 | Menthol, Clove, Onion, Gingerbread, Orange |
| 6 | 0 | 16.9 | 98.5 | 68.6 | 86.2 | Pizza, Turpentine, Root beer, Lime, Grass, Grape |
| 6 | 1 | 44.4 | 94.8 | 61.8 | 90.0 | Pizza, Cherry, Root beer, Dill pickle, Grass, Grape |
| 6 | 2 | 71.1 | 85.8 | 48.8 | 94.0 | Pizza, Cherry, Strawberry, Turpentine, Root beer, Dill pickle |
| 6 | 3 | 83.8 | 82.6 | 47.8 | 96.4 | Pizza, Clove, Fruit Punch, Liquorice, Lime, Pine |
| 6 | 4 | 84.5 | 83.8 | 49.8 | 96.6 | Menthol, Cherry, Clove, Gingerbread, Root beer, Orange |
| 6 | 5 | 83.8 | 80.9 | 45.4 | 96.3 | Menthol, Clove, Gingerbread, Lilac, Watermelon, Smoke |
| 7 | 0 | 11.3 | 99.2 | 72.7 | 85.5 | Pizza, Turpentine, Root beer, Dill pickle, Lime, Grass, Grape |
| 7 | 1 | 37.3 | 96.4 | 66.3 | 89.0 | Pizza, Cherry, Root beer, Dill pickle, Lime, Grass, Grape |
| 7 | 2 | 69.0 | 85.7 | 47.8 | 93.6 | Pizza, Fruit Punch, Strawberry, Turpentine, Root beer, Dill pickle, Lime |
| 7 | 3 | 78.2 | 86.8 | 52.9 | 95.4 | Pizza, Fruit Punch, Strawberry, Root beer, Dill pickle, Lemon, Rose |
| 7 | 4 | 82.4 | 87.7 | 56.0 | 96.3 | Cherry, Mint, Clove, Fruit Punch, Gingerbread, Root beer, Pine |
| 7 | 5 | 87.3 | 83.0 | 49.4 | 97.2 | Pizza, Menthol, Cherry, Clove, Gingerbread, Orange, Pine |
| 7 | 6 | 84.5 | 79.7 | 44.1 | 96.4 | Menthol, Clove, Leather, Lilac, Watermelon, Smoke, Rose |

**+ve = positive hyposmia threshold cut off*

**Supplementary Table 5:**

| **Number of smells** | **+ve* cutoff** | **Sensitivity** | **Specificity** | **PPV** | **NPV** | **Smells** |
| --- | --- | --- | --- | --- | --- | --- |
| 1 | 0 | 82.4 | 53.5 | 27.7 | 93.3 | Pizza |
| 2 | 0 | 50.0 | 82.2 | 37.8 | 88.4 | Pizza, Root beer |
| 2 | 1 | 61.8 | 82.8 | 43.8 | 90.9 | Clove, Coconut |
| 3 | 0 | 38.2 | 90.4 | 46.4 | 87.1 | Pizza, Root beer, Dill pickle |
| 3 | 1 | 70.6 | 80.9 | 44.4 | 92.7 | Pizza, Clove, Root beer |
| 3 | 2 | 70.6 | 71.3 | 34.8 | 91.8 | Menthol, Clove, Coconut |
| 4 | 0 | 14.7 | 96.8 | 50.0 | 84.0 | Pizza, Root beer, Lime, Grape |
| 4 | 1 | 55.9 | 90.4 | 55.9 | 90.4 | Pizza, Cherry, Turpentine, Root beer |
| 4 | 2 | 61.8 | 88.5 | 53.8 | 91.4 | Cherry, Clove, Coconut, Root beer |
| 4 | 3 | 91.2 | 35.0 | 23.3 | 94.8 | Menthol, Clove, Gingerbread, Orange |
| 5 | 0 | 5.9 | 99.4 | 66.7 | 83.0 | Pizza, Root beer, Lime, Grass, Grape |
| 5 | 1 | 35.3 | 95.5 | 63.2 | 87.2 | Pizza, Cherry, Turpentine, Root beer, Lime |
| 5 | 2 | 73.5 | 84.1 | 50.0 | 93.6 | Pizza, Clove, Fruit Punch, Chocolate, Root beer |
| 5 | 3 | 67.6 | 82.8 | 46.0 | 92.2 | Menthol, Cherry, Clove, Coconut, Root beer |
| 5 | 4 | 94.1 | 33.8 | 23.5 | 96.4 | Menthol, Clove, Onion, Gingerbread, Orange |
| 6 | 0 | 2.9 | 100.0 | 100.0 | 82.6 | Pizza, Turpentine, Root beer, Lime, Grass, Grape |
| 6 | 1 | 32.4 | 99.4 | 91.7 | 87.2 | Pizza, Cherry, Root beer, Dill pickle, Grass, Grape |
| 6 | 2 | 52.9 | 93.6 | 64.3 | 90.2 | Pizza, Cherry, Strawberry, Turpentine, Root beer, Dill pickle |
| 6 | 3 | 85.3 | 78.3 | 46.0 | 96.1 | Pizza, Clove, Fruit Punch, Liquorice, Lime, Pine |
| 6 | 4 | 88.2 | 65.6 | 35.7 | 96.3 | Menthol, Cherry, Clove, Gingerbread, Root beer, Orange |
| 6 | 5 | 85.3 | 68.2 | 36.7 | 95.5 | Menthol, Clove, Gingerbread, Lilac, Watermelon, Smoke |
| 7 | 0 | 0.0 | 100.0 | 100.0 | 82.2 | Pizza, Turpentine, Root beer, Dill pickle, Lime, Grass, Grape |
| 7 | 1 | 17.6 | 99.4 | 85.7 | 84.8 | Pizza, Cherry, Root beer, Dill pickle, Lime, Grass, Grape |
| 7 | 2 | 47.1 | 91.1 | 53.3 | 88.8 | Pizza, Fruit Punch, Strawberry, Turpentine, Root beer, Dill pickle, Lime |
| 7 | 3 | 67.6 | 83.4 | 46.9 | 92.3 | Pizza, Fruit Punch, Strawberry, Root beer, Dill pickle, Lemon, Rose |
| 7 | 4 | 88.2 | 85.4 | 56.6 | 97.1 | Cherry, Mint, Clove, Fruit Punch, Gingerbread, Root beer, Pine |
| 7 | 5 | 100.0 | 53.5 | 31.8 | 100.0 | Pizza, Menthol, Cherry, Clove, Gingerbread, Orange, Pine |
| 7 | 6 | 85.3 | 58.0 | 30.5 | 94.8 | Menthol, Clove, Leather, Lilac, Watermelon, Smoke, Rose |

**+ve = positive hyposmia threshold cut off*

**Supplementary Table 6:** Differing distractor options used for specific smells included within shorted PST Versions and B, and those for the same smells within the full UPSIT test

| **Correct smell** | **PST Version** | **PST Distractor options** | **UPSIT distractor options** |
| --- | --- | --- | --- |
| Chocolate | A | Lemon, *smoke*, black pepper | Lemon, *root beer*, black pepper |
| Strawberry | A | Garlic, leather, gasoline | Dill pickle, chocolate, cedar |
| Smoke | A | *Garlic*, grass, peach | *Dill pickle*, grass, peach |
| Leather | A | *Mint, a flower*, apple | *Clove, lilac*, apple |
| Soap | B | Black pepper, *leather*, peanut | Black pepper, *liquorice*, peanut |
| Grape | B | *Gasoline, rose, peanut* | *Pizza, turpentine, clove* |
| Onion | B | Chocolate, *strawberry*, fruit punch | Chocolate, *banana*, fruit punch |
| Natural gas | B | Orange, *cinnamon*, cola | Orange, *musk*, cola |

*Items in *italics* are distractor options which differ between UPSIT and PST versions

**Supplementary Table 7: Screening performance of “winning” smell subsets with UK compatible UPSIT smells, from *discovery* cohort and re-assessment in *validation* cohort**

| **No. of smells** | **Hyposmia cut-off score** | **Smells “winning” in discovery cohort** | **Sens. in discovery cohort** | **Spec. in discovery cohort** | **PPV in discovery cohort** | **NPV in discovery cohort** | **Sens.in validation cohort** | **Spec. in validation cohort** | **PPV in validation cohort** | **NPV in validation cohort** |
| --- | --- | --- | --- | --- | --- | --- | --- | --- | --- | --- |
| 1 | 0 | Pizza | 69.7 | 66.1 | 28.0 | 92.0 | 82.4 | 53.5 | 27.7 | 93.3 |
| 2 | ≤1 | Clove, Coconut | 62.0 | 88.7 | 50.9 | 92.5 | 61.8 | 82.8 | 43.8 | 90.9 |
| 3 | ≤2 | Menthol, Clove, Coconut | 71.8 | 86.8 | 50.7 | 94.2 | 70.6 | 71.3 | 34.8 | 91.8 |
| 4 | ≤3 | Menthol, Clove, Gingerbread, Orange | 78.9 | 82.8 | 46.5 | 95.4 | 91.2 | 35.0 | 23.3 | 94.8 |
| 5 | ≤4 | Menthol, Clove, Onion, Gingerbread, Orange | 83.1 | 80.4 | 44.5 | 96.2 | 94.1 | 33.8 | 23.5 | 96.4 |
| 6 | ≤5 | Menthol, Clove, Gingerbread, Lilac, Watermelon, Smoke | 83.8 | 80.9 | 45.4 | 96.3 | 85.3 | 68.2 | 36.7 | 95.5 |
| 7 | ≤5 | Pizza, Menthol, Cherry, Clove, Gingerbread, Orange, Pine | 87.3 | 83.0 | 49.4 | 97.2 | 100.0 | 53.5 | 31.8 | 100.0 |
| 7 | ≤6 | Menthol, Clove, Leather, Lilac, Watermelon, Smoke, Rose | 84.5 | 79.7 | 44.1 | 96.4 | 85.3 | 58.0 | 30.5 | 94.8 |
